# Impact of Heterogeneity and Secrecy on theCapacity of Wireless Sensor Networks

**DOI:** 10.3390/s151229844

**Published:** 2015-12-10

**Authors:** Qiuming Liu, Li Yu, Zuhao Liu, Jun Zheng

**Affiliations:** 1 School of Electronic Information and Communications, Huazhong University of Science and Technology, 1037 Luoyu Road, Wuhan 430074, China; liuqiuming@hust.edu.cn (Q.L.); junzheng@hust.edu.cn (J.Z.); 2 Nanchang of Jiangxi University of Science and Technology, 1180 Shuanggang Road, Nanchang 330013, China; 3 China Yangtze Power Co., Ltd., 1 Xibajianshe Road, Yichang 443002, China; liuzuhao@gmail.cn

**Keywords:** secrecy throughput, percolation, heterogeneous topology, wireless sensor networks

## Abstract

This paper investigates the achievable secrecy throughput of an inhomogeneous wireless sensor network. We consider the impact of topology heterogeneity and the secrecy constraint on the throughput. For the topology heterogeneity, by virtue of percolation theory, a set of connected highways and information pipelines is established; while for the secrecy constraint, the concept of secrecy zone is adopted to ensure secrecy transmission. The secrecy zone means there is no eavesdropper around the legitimate node. The results demonstrate that, if the eavesdropper’s intensity is $\lambda_{e}=o\left({\left(\log n\right)}^{-\frac{3\delta -4}{\delta -2}}\right)$, a per-node secrecy rate of $\Omega \left(\frac{1}{\sqrt{n^{1-v}\left(1-v\right)\log n}}\right)$ can be achieved on the highways, where *δ* is the exponent of heterogeneity, *n* and $n^{v}$ represent the number of nodes and clusters in the network, respectively. It is also shown that, with the density of the eavesdropper $\lambda_{e}=o\left({\left(\log\left(n\underline{\Phi }\right)\right)}^{-2}\right)$, the per-node secrecy rate of $\Omega \left(\sqrt{\frac{\underline{\Phi }}{n}}\right)$ can be obtained in the information pipelines, where $\underline{\Phi }$ denotes the minimum node density in the network.

## 1. Introduction

Wireless sensor networks are an emerging networking technology, which is widely used in environmental monitoring, emergency and rescue communication, military applications, *etc.* The unique feature of such networks is formed by the huge number of sensor nodes. Each node communicates over a wireless channel without any centralized control [1]. One of the problems in wireless sensor network is efficient data transmission and lifetime. The low-energy adaptive clustering hierarchy (LEACH) protocol presented by Heinzelman *et al.* [2] was a widely known and effective one to reduce and balance the total energy consumption. Later, Tan *et al.* [3] proposed an energy-efficient hybrid cluster-based protocol (HCEP) to prolong the lifetime of the network. To reduce the consumption of energy, Wu *et al.* [4] developed a structure fidelity data collection (SFDC) framework to reduce the number of active sensor nodes, which can not only save energy, but also reserve the data fidelity. Another problem is the throughput capacity, meaning how much traffic the wireless networks can carry. In their groundbreaking work, Gupta and Kumar [5] had shown that, for a static wireless networks consisting of *n* nodes randomly and uniformly distributed, each node can achieve a rate of order $\Omega \left(\frac{1}{\sqrt{n\log n}}\right)$. Given two functions f(n) and g(n): f(n)=o(g(n)) means $\lim_{n\to \infty }f\left(n\right)/g\left(n\right)=0$; f(n)=O(g(n)) means $\lim_{n\to \infty }f\left(n\right)/g\left(n\right)=c<\infty$; if g(n)=O(f(n)), f(n)=Ω(g(n)) w.h.p.; if both f(n)=Ω(g(n)) and f(n)=O(g(n)), f(n)=Θ(g(n)); $f\left(n\right)=\tilde{\Theta }\left(g\left(n\right)\right)$ means f(n)=Θ(g(n)) when logarithmic terms are ignored. They also derived an upper bound on the capacity that scaled on the order $O\left(\frac{1}{\sqrt{n}}\right)$. This capacity gap was closed by Franceschetti *et al.* [6]. Inspired by percolation theory, they constructed a series of paths spanning the network both horizontally and vertically. Then, by exploiting the time division multiple access (TDMA) strategy, each node transmitted its information to the nearest horizontal highway. After that, the information was transported in a multi-hop manner toward the vertical paths, which was near the receiver. Finally, the information was sent to the receiver from the existing node on the vertical highway. Based on the “highways scheme”, a rate of $\Theta \left(\frac{1}{\sqrt{n}}\right)$ was achieved for each node. Since then, capacity scaling has drawn considerable attention. Hu *et al.* [7] investigated the impact of geometry on the capacity of a wireless network. They constructed highways in a strip network, triangle network and three-dimensional network. Since the infrastructure was an effective way to ease hop-by-hop transmission, Liu *et al.* [8] allocated some infrastructure into the network and proved that the capacity could increase linearly with the number of infrastructures. Tan *et al.* [9] proposed a framework to maximize the total utility of bandwidth allocation for the three traffic types in infrastructure-based wireless networks. Multicast was often used in realistic networks; Li [10] derived the multicast capacity of large-scale wireless networks using a tree-based routing scheme. Later on, Alfano *et al.* [11,12] firstly investigated the capacity of topology inhomogeneous wireless networks. Liu *et al.* [13] constructed a “highway system” in inhomogeneous Poisson networks. Based on the highway system, the lower bound of capacity was obtained, and they found that the bottleneckof the rate was caused by the place of the lowest node density. After that, the scenario of traffic heterogeneity was studied. Kim *et al.* [14] proposed a differentiated channel access scheme to resolve the throughput fairness problem in heterogeneous wireless networks. Recently, Lu and Shen [15] gave a comprehensive overview of the development of capacity and delay in *ad hoc* networks. They also presented the fundamental tradeoffs between capacity and delay under a variety of mobility models.

However, due to the wireless channel being broadcast, it is easily attacked by eavesdroppers and malicious nodes. This motivates considering the secrecy constraint in capacity analysis. With some exceptions, the secrecy capacity under the protection of an RSApublic key cryptosystem was used in [16,17]. They got a pessimistic result that, for a network consisting of *n* legitimate nodes, a rate of $\Omega \left(\sqrt{\frac{p_{f}}{n\log n}}\right)$ was obtained, where $p_{f}$ was the probability that a node shared a primary secure association (SA) with any other node. To avoid the capacity degradation caused by $p_{f}$ decreasing, an information theoretic security was proposed, which was achieved by using the channel difference between legitimate nodes and eavesdroppers, which required the intended receiver to have a stronger channel than eavesdroppers. To degrade the signal of eavesdropper, Vasudevan *et al.* [18] used other nodes around the transmitters to generate artificial noise. They found that, when a per-node throughput of $\Omega \left(\frac{1}{\sqrt{n\log n}}\right)$ was desired, the network can tolerate up to $\Omega \left({\left(\log n\right)}^{c}\right)$ independent eavesdroppers or a single eavesdropper with $\Omega \left({\left(\log\log n\right)}^{1-\epsilon }\right)$ antennas, where *c* and *ϵ* were constants. After that, Capar *et al.* [19] investigated the impact of network dimension on the secrecy capacity. They found that the per-node secure throughput was $\Omega \left(\frac{1}{n}\right)$ in one-dimensional and $\Omega \left(\frac{1}{\sqrt{n\log n}}\right)$ in two-dimensional networks, respectively. More recently, Zhang *et al.* [20] considered a homogeneous network with an independent eavesdropper and colluding eavesdroppers. Each node was installed with three antennas, where two of them were used for transmitting and receiving, and the other one was employed to generate artificial noise to degrade the signal of the eavesdropper. By constructing a set of highways in the networks, they derived that the secrecy capacity was $\Theta \left(\frac{1}{\sqrt{n}}\right)$ for the scenario of an independent and colluding eavesdropper. Later on, Cao *et al.* [21] investigated the tradeoff between secrecy capacity and delay in large-scale mobile *ad hoc* networks. They found that, for a given delay constraint *D*, the optimal secrecy throughput capacity was $\tilde{\Theta }\left({\left(\frac{D}{n}\right)}^{\frac{2}{3}}\right)$. In addition to the method of generating artificial noise to suppress the eavesdroppers’ receiving signal, an alternative idea of the secrecy zone was proposed in [22,23], which required neither the channel state information of eavesdroppers, nor extra power to generate artificial noise. Under the protection of the secrecy zone, Koyluoglu *et al.* [22] obtained that, as long as the density of the eavesdropper was $o\left(\frac{1}{{\left(\log n\right)}^{2}}\right)$, each node can achieve a secure rate of $\Omega \left(\frac{1}{\sqrt{n}}\right)$. Besides the information security issue in wireless networks, privacy security is also concerned. To avoid disclosing the users’ interest to others, Luan and Lu *et al.* [24] employed a privacy-preserving mechanism to protect sensitive user information during social communications. Although there existed many works on security issues, all of them focused on homogeneous networks. Therefore, a fundamental question arises: what is the impact of the capacity if both the security constraint and heterogeneity topology are taken into consideration?

In this paper, we focus on static cluster sparse networks. Our main purpose is solving the secrecy transmission in heterogeneity networks. The transmission is divided into intra-cluster and inter-cluster traffic. For the former transmission, we propose a heterogeneous percolation model. Based on the heterogeneous percolation, we construct a series of “paths” in the radial direction and around the cluster. The information is transported in a multi-hop manner on the paths. While for the latter traffic, some information pipelines have been built among clusters. On the basis of the “highway system” and information pipelines, we employ the secrecy zone to protect the transmission. This is different from the artificial noise generation fashion, where their strategy needs additional power to generate noise.

The main contributions can be concluded as follows:

- We prove the existence of highways in heterogeneous networks. More importantly, it is shown that the networks still percolate in the secrecy constraint model, and many secrecy highway paths can be constructed.
- We first exploit the secrecy zone to protect the transmission in heterogeneous networks. The relationship between the secrecy capacity and the tolerable density of the eavesdropper was established.
- Due to the impact of heterogeneity, we observe that the secrecy throughput of intra-cluster transmission is higher than that in a homogeneous one, and the bottleneck of secrecy throughput is located at the area with the minimum node density.

The rest of this paper is organized as follows. In Section 2, we give the network model. We give the transmission model in Section 3. The construction of the circular percolation model is described in Section 4. Section 5 derives the secrecy throughput in intra-cluster transmission. In Section 6, we investigate the secrecy throughput of inter-cluster transmission. We present the conclusion of this paper in Section 7.

## 2. Network Model

We consider an extended network $A=\left[0,\sqrt{n}\right]\times \left[0,\sqrt{n}\right]$ with *n* legitimate nodes randomly distributed, where the distribution of legitimate nodes follows the shot noise Cox process (SNCP) [25]. The main process of SNCP is described as follows: *M* clusters scattered in *A* randomly. The expected value of *M* is E(M)=m. We denote the center of the clusters as $C={\left\{c_{j}\right\}}_{j=1}^{M}$. For each $c_{j}$, using the center point $c_{j}$ as a mother point, a point process centered by $c_{j}$ with an intensity of $q_{j}k\left(c_{j},\xi \right)$ at place *ξ* is generated. $k(c_{j},\xi )$ is a function of density; $q_{j}$ is the number of nodes of cluster $c_{j}$. Let each cluster consist of an equal number of legitimate nodes, *i.e.*, $q_{j}=n/m$. In addition, according to the distribution, the function of density *F* at place *ξ* can be expressed as:

(1) $$F\left(\xi \right)=\sum_{j}q_{j}k\left(c_{j},\xi \right)$$

where $k\left(c_{j},\xi \right)=k(||\xi -c_{j}\left||\right)$ is related to the distance between *ξ* and $c_{j}$, and the sum $\int_{A}k\left(c_{j},\xi \right)d\xi$ on the whole network is finite. For simplicity, we use function s(ρ) to substitute the density function $k\left(c_{j},\xi \right)$, where $\rho =||\xi -c_{j}\left|\right|$. To gain finite summation over the whole area, the function s(ρ) is stated as follows:

(2) $$s\left(\rho \right)=\min\left(1,\rho^{-\delta }\right),\delta >2$$

In addition, let *m* scale as $\Theta (n^{v})$; let *δ* be a degradation factor; and v∈(0,1). Then, each cluster has a number of nodes $\Theta \left(n^{1-v}\right)$, *i.e.*, $q_{j}=\Theta \left(n^{1-v}\right)$ for j=1,2,⋯,M, since $\int_{A}k\left(c_{j},\xi \right)\text{d}\xi$ is finite.

According to the node’s distribution, we can obtain the average distance between each cluster center $d_{c}$ as:

(3) $$d_{c}=\Theta \left(\sqrt{\frac{A}{m}}\right)=\Theta \left(n^{\frac{1-v}{2}}\right)$$

**Figure 1:**
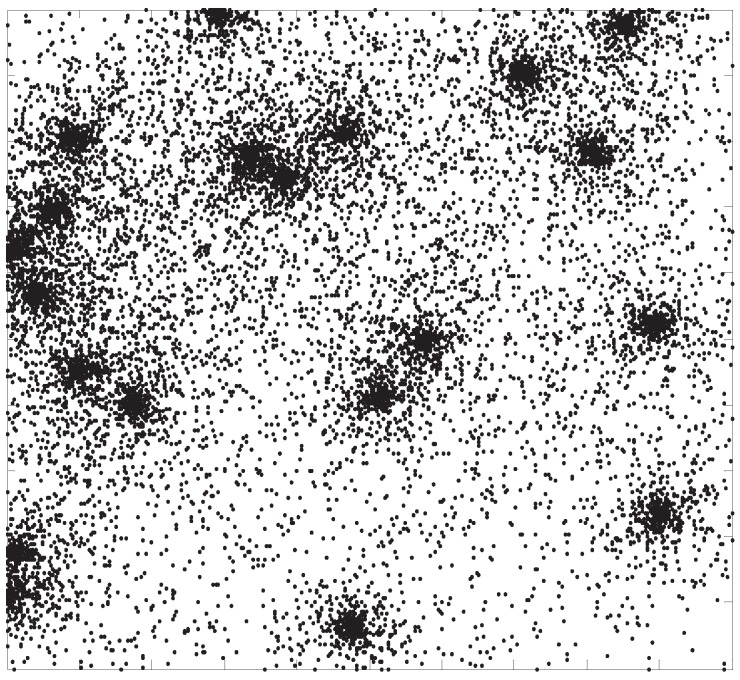
Example of a heterogeneous topology network. The parameter of the network is n= 10,000, v=0.3 and δ=3.

From Equation (3), we know, when v<1, $d_{c}\to \infty$ as n→∞, and the clusters are distributed sparsely. In this work, we only consider the cluster-sparse network, where the s(ρ) is heterogeneous. Let $\bar{\Phi }$ denote the largest density and $\underline{\Phi }$ *vice versa*. Figure 1 is an example of this kind of network topology.

Different from the legitimate nodes, the eavesdroppers are uniformly and independently distributed in the network with density $\lambda_{e}$. Let *ε* denote the set of eavesdroppers. Since eavesdroppers can be easily detected if they are active, the eavesdroppers are assumed to keep silent. To get insight into the secrecy throughput, the eavesdroppers is also assumed to have a super ability for computing. This means that the traditional method cannot be used here. In addition, let the transmitters know the location of the eavesdropper. Although the assumption seems idealistic, it allows one to gain valuable insight into the problem.

To get the worst case of secrecy throughput, the interference caused by simultaneous transmission is assumed as noise, whereas the eavesdroppers do not have this assumption.

## 3. Transmission Model

For random chosen S-Dpairs, the transmitter *i* wants to send the information $W_{i,j}$ to a receiver *j* securely. During time slot *t*, let signals observed at eavesdropper *e* be $Y_{e}≜Y_{e}\left(t\right),\forall t$. In the multi-hop routing, each session in one hop has *N* channels. Let *R* be the achievable secrecy rate for the S-D pairs (i,j), if:

- The error decoding the probability of the transmission information at the receiver can be treated arbitrarily low as N→∞.
- The leakage rate information associated with the transporting information over the whole path, *i.e*., $\frac{I(W_{i,j};Y_{e})}{N}$, goes to arbitrarily small ∀e∈ε as N→∞.

For almost all (i,j), if the message $W_{i,j}$ is transmitted within *H* hops, we only need to observe the channel of the eavesdropper when considering the security. Hence, the second condition can be satisfied if $\frac{I(W_{i,j};Y_{e}\left(1\right),,Y_{e}\left(H\right))}{N}$ can be made arbitrarily small if the block length is sufficiently large, where $Y_{e}\left(h\right)$ denotes the output vector of length *N* at eavesdropper e∈ε during hop *h*.

We consider the Gaussian wiretap channel capacity [26]. Let $SINR_{ij}$ be the signal-to-interference and noise ratio (SINR) from legitimate transmission node *i* to legitimate destination node *j* over a channel of unit bandwidth, which is given as:

(4) $$SINR_{ij}=\frac{P_{i}l\left(i,j\right)}{N_{0}+\sum_{\zeta \in T\backslash \{i\}}P_{\zeta }l\left(\zeta ,j\right)}$$

where l(i,j) = min$\left\{1,1/d_{ij}^{\alpha }\right\}$ with α>2 representing the path loss of the channel between node *i* and node *j*. $P_{i}$ is the power of transmitting node *i*. $N_{0}$ denotes the noise power at the receiving node *j*, and *ζ* represents the set of nodes that can transmit simultaneously with node *i*.

Similarly, the SINR received at eavesdropper *e* is as follows:

(5) $$SINR_{ie}=\frac{P_{i}l\left(i,e\right)}{N_{0}+\sum_{\zeta \in T}P_{\zeta }l\left(\zeta ,e\right)}$$

According to the secrecy throughput defined in [26,27], the secure rate of any legitimate node can be denoted as:

(6) $$R_{ij}^{s}=R_{ij}-\bar{R_{ie}}=\log_{2}\left(1+SINR_{ij}\right)-\log_{2}\left(1+\bar{SINR_{ie}}\right)$$

where $\bar{SINR_{ie}}=\max_{e\in \varepsilon }SINR_{ie}$.

Due to the impact of heterogeneity, we use different powers for different nodes. The secrecy rate is defined as $R_{s}\left(n\right)$, which is also the maximum achievable secrecy capacity.

## 4. Circular Percolation

We first construct a percolation model for the heterogeneous topology. Since the legitimate nodes are distributed heterogeneously, the traffic is divided into two parts: intra-cluster traffic and inter-cluster traffic. For each part of the traffic, we resort to the tools of percolation theory to construct the routing scheme. For the intra-cluster traffic, we establish a circle percolation model, which is different from the previous percolation model; while for the inter-cluster case, we construct a series of information pipelines to link the clusters.

In our model, by virtue of percolation theory, we present a circular percolation model and construct a set of connected highways for legitimate nodes. Different from the the work in [6], the circular highways are from internal to external or encircling the cluster.

**Lemma 1.** *Assume $\rho_{\min}$ is a minimum positive constant that separates the $c_{j}$ and other nodes. Then, each cluster can build a crossing path within $\rho_{\max}$ for δ>2.*

**Figure 2:**
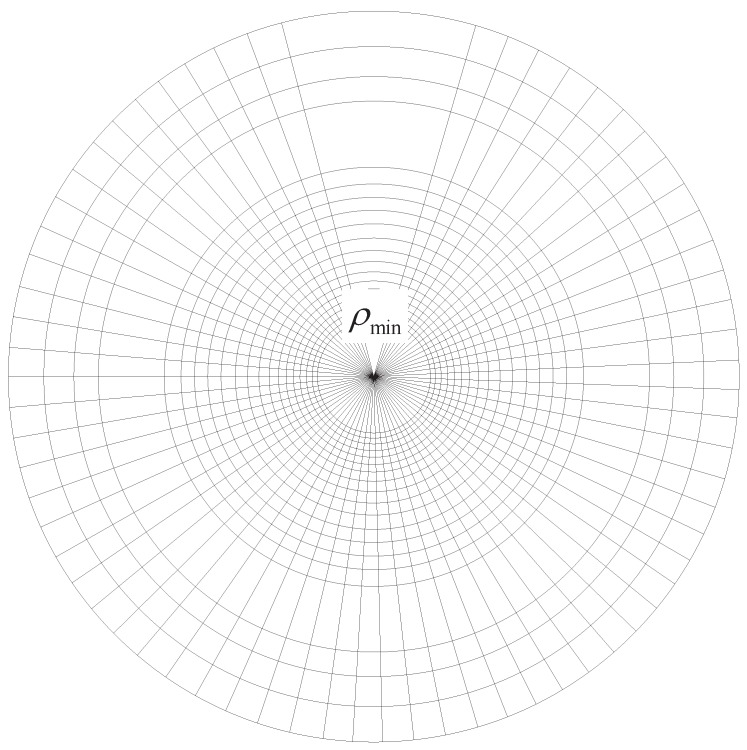
A circular square in a cluster. $\rho_{\min}$ is the minimum radius within which there is no node located.

**Proof.** Each cluster is tessellated into $x\times \frac{n/m}{x}$ circular squares, where *x* is the number of sectors and $\frac{n/m}{x}$ is the number of annuli. Note that the arc of each sector is equal, *i.e*., $\frac{2\pi }{x}$. Due to the heterogeneous node distribution, the distance between every two annuli is not the same, as shown in Figure 2.

Due to the impact of heterogeneity, the node density at different annulus areas varies. In order to guarantee that each square contains at least one node, the external annuli need to be wider than the inner ones. Correspondingly, we set the radius of the *i*-th annulus to be:

(7) $$r_{i}={\left(1+\frac{2\pi }{x}\right)}^{i-1}\cdot \rho_{\min}$$

Thus, according to the circular square tessellation above, we can conclude that each cell can be treated as a square when n→∞. ☐

**Lemma 2.** *According to the heterogeneous tessellation, we can get that the number of the parameter x is: $x=\Theta \left(\sqrt{\frac{n^{1-v}}{(1-v)\log n}}\right)$.*

**Proof.** From Lemma 1, we know that the radius of one cluster is:

(8) $$r_{\frac{n/m}{x}+1}={\left(1+\frac{2\pi }{x}\right)}^{\frac{n/m}{x}},\;\;n\to \infty$$

Combining Equation (3) and Equation (8), we have:

(9) $$r_{\frac{n/m}{x}+1}={\left(1+\frac{2\pi }{x}\right)}^{\frac{n/m}{x}}=e^{\frac{2\pi n^{1-v}}{x^{2}}}=\frac{d_{c}}{2}$$

Finally, we obtain $x=\Theta \left(\sqrt{\frac{n^{1-v}}{(1-v)\log n}}\right)$. ☐

**Lemma 3.** *For a square $s_{i}$ on the i-th annulus, let $X_{s_{i}}$ be the number of nodes distributed in $s_{i}$; then, we can get $P\left(X_{s_{i}}\geq 1\right)>P\left(X_{s_{j}}\geq 1\right)$ for i<j.*

**Proof.** According to percolation theory, a square $s_{i}$ is open if there exists at least one node located in $s_{i}$, or closed otherwise. From the Appendix A in [12], the open probability of a square is:

(10) $$\begin{array}{cc} p_{i}\equiv P\left(X_{s_{i}}\geq 1\right) & =1-P\left(X_{s_{i}}=0\right)\\ & \approx 1-e^{-\frac{n}{m}r_{i}^{-\delta }{\left(r_{i}\frac{2\pi }{x}\right)}^{2}} \end{array}$$

From Equation (10), we can observe that $p_{i}$ decreases as increasing values of *i* for δ>2. ☐

Since the probability $p_{i}$ decreases with *i*, we can calculate the critical value $\rho_{\max}$, within which the probability $p_{i}$ can satisfy the condition of $p_{i}>p_{c}$, where $p_{c}$ is the critical probability in percolation theory.

**Lemma 4.** *There exists a critical value $\rho_{\max}=\Theta \left({\left((1-v)\log n\right)}^{\frac{1}{\delta -2}}\right)$, within which each cluster can construct a set of connected highways if the degradation factor δ>2.*

**Proof.** From percolation theory, there is a critical probability $p_{c}$, when $p>p_{c}$, that there exists many disjoint paths traversing the network, which is going to be one. Thus, we can get the following equation by Lemma 3:

(11) $$p_{i}\equiv P\left(X_{s_{i}}\geq 1\right)\approx 1-e^{-{r_{i}}^{-\delta }{\left(r_{i}\frac{2\pi }{x}\right)}^{2}}=p_{o}$$

where $p_{o}$ is the probability representing that the square $s_{i}$ is open, and $p_{c}<p_{o}<1$.

Following Equation (11), we can obtain that:

(12) $$\frac{n}{m}{r_{i}}^{-\delta }{\left(r_{i}\frac{2\pi }{x}\right)}^{2}=c$$

where $c=\ln\frac{1}{1-p_{o}}$. Substituting $x=\Theta \left(\sqrt{\frac{n^{1-v}}{(1-v)\log n}}\right)$ into (12), we can achieve that $\rho_{\max}=\Theta \left({\left((1-v)\log n\right)}^{\frac{1}{\delta -2}}\right)$. ☐

Combining Lemma 4 and Equation (7), the critical value $i_{\max}=\Theta \left(\frac{\log(\log n)}{\delta -2}\sqrt{\frac{n^{1-v}}{\log n}}\right)$ is achieved. In particular, we can construct $\Theta \left(\frac{\log(\log n)}{\delta -2}\sqrt{\frac{n^{1-v}}{\log n}}\right)$ annuli within the radius $\rho_{\max}$.

The percolation model for the intra-cluster traffic has been constructed. Each cluster is partitioned into $c_{1}\sqrt{\frac{n^{1-v}}{(1-v)\log n}}\times c_{2}\frac{\log(\log n)}{\delta -2}\sqrt{\frac{n^{1-v}}{\log n}}$ lattices. Specifically, a path is called open if two adjacent squares are open. Based on Appendix I in [6], we can get that there are ⌈μlogω(n)⌉ disjoint paths through an area of ω(n)×(κlogω(n)-ϵ). Hence, within area of less than $\rho_{\max}$, we can build $\Omega \left(\sqrt{\frac{n^{1-v}}{(1-v)\log n}}\right)$ disjoint paths from the internal to external cluster and $\Omega \left(\frac{\log(\log n)}{\delta -2}\sqrt{\frac{n^{1-v}}{\log n}}\right)$ disjoint paths around each cluster. By the connection of these two paths, the highway system for the legitimate nodes is constructed. Although our model is a heterogeneous lattice, it can be treated similarly as a $c_{1}\sqrt{\frac{n^{1-v}}{(1-v)\log n}}\times c_{2}\frac{\log(\log n)}{\delta -2}\sqrt{\frac{n^{1-v}}{\log n}}$ rectangle lattice.

## 5. The Secrecy Rate of Intra-Cluster Traffic

**Figure 3:**
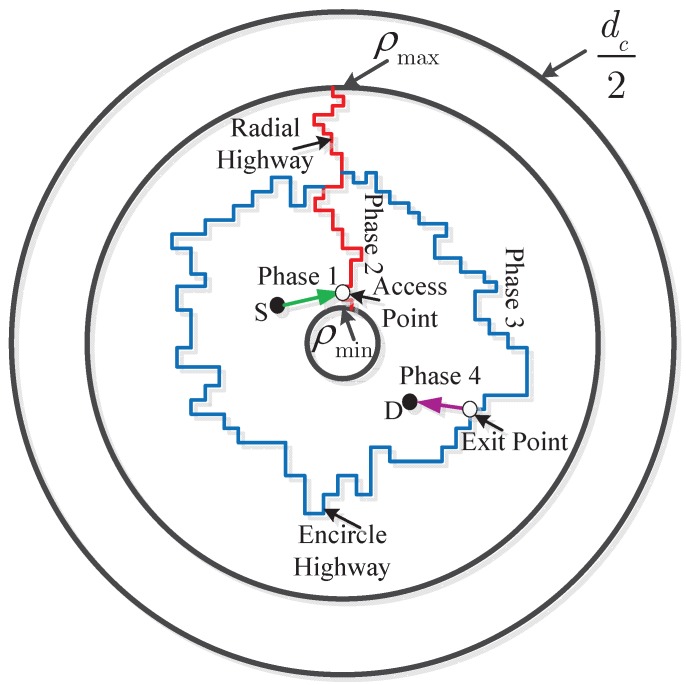
A schematic representation of the routing scheme. We omit the eavesdroppers in this figure.

In this section, as illustrated in Figure 3, we introduce a scheme that ensures the security over the whole path, from the source to a destination. The routing scheme of intra-cluster transmission can be partitioned into four separate phases.

Phase 1 (draining phase): Source node *S* drains packets to an access node on the radial highway directly. Note that the highway may not be fully contained in its corresponding sector, whereas it may deviate from it. However, according to percolation theory, a highway is never farther than $\kappa \log\left(\frac{n/m}{x}-\epsilon \right)$ from its corresponding sector.

Phase 2 (radial highway phase): Packets are carried across the cluster along the radial highway using multiple hops and multiple time slots.

Phase 3 (encircling highway phase): Similar to Phase 2, packets are transported clockwise on the annulus highway.

Phase 4 (delivering phase): Finally, packets are delivered to the receiver from the exit point on the encircling highway.

Different nodes are allocated with different power, so that we can transform the heterogeneous circular lattice into a homogenous regular square lattice. In addition, the time division multiple access (TDMA) schedule is employed, and the information is transported hop by hop, then a constant rate on the highway is obtained. However, there are still some differences between our model and the previous one; for example, not all of the highways serve identical nodes, and the power of each node is not equal.

**Lemma 5.** *For a given square, to cancel the interference caused by simultaneous transmission, the power of legitimate nodes in square $s_{i}$ is:*

(13) $$P_{i}=P_{0}\cdot {\left(\frac{2\pi r_{i}}{x}\right)}^{\alpha }$$

*where $P_{0}$ is the unit power for a legitimate transmitter.*

**Proof.** For a square $s_{i}$, let $I_{1}$ be the interference from the outside square and $I_{2}$ for that from the inside square. If the distance between two nodes is *d*, which is not the Euclidean distance, but the number of *d* squares away, then we can get the interference from different directions as follows:

(14) $$I_{1}\left(d\right)=P_{\left(i+d\right)}\frac{1}{{\left(r_{i+d}-r_{i}\right)}^{\alpha }}$$

(15) $$I_{2}\left(d\right)=P_{\left(i-d\right)}\frac{1}{{\left(r_{i}-r_{i-d}\right)}^{\alpha }}$$

We can also get that:

(16) $$\begin{array}{cc} \frac{I_{1}\left(d\right)}{I_{2}\left(d\right)} & =\frac{P_{\left(i+d\right)}}{P_{\left(i-d\right)}}\cdot \frac{{\left(r_{i}-r_{i-d}\right)}^{\alpha }}{{\left(r_{i+d}-r_{i}\right)}^{\alpha }}\\ & ={\left(\frac{r_{i+d}}{r_{i-d}}\cdot \frac{r_{i}-r_{i-d}}{r_{i+d}-r_{i}}\right)}^{\alpha }\\ & ={\left(1+\frac{2\pi }{x}\right)}^{d\alpha } \end{array}$$

As $x=\Theta \left(\sqrt{\frac{n^{1-v}}{(1-v)\log n}}\right)$, when $d=o\left(\sqrt{\frac{n^{1-v}}{(1-v)\log n}}\right)$, we can get $\frac{I_{1}\left(d\right)}{{\;I}_{2}\left(d\right)}\to 1$. ☐

Next, we exploit the idea of the secrecy zone to guarantee the secrecy of the communication over a single hop.

**Figure 4:**
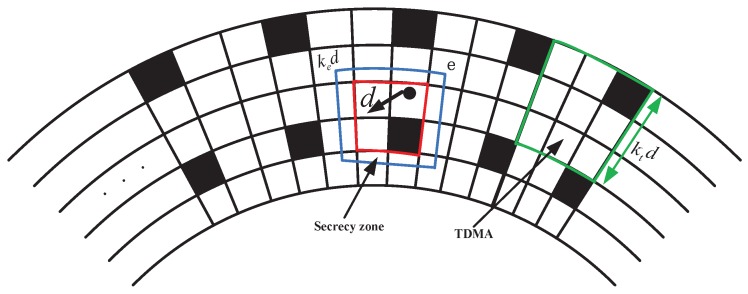
An illustration of the TDMA strategy with size $k_{t}d$. The blue square surrounding the transmitter is the secrecy zone, which is at most $k_{e}d$ squares away from the transmitter.

By Lemma 5, for a given square, the interference caused at *d* squares is equal. Thus, we can make the cluster network as a square network. As shown in Figure 4, we group several squares into a group with edge $k_{t}d$ squares. Each group contains ${\left(k_{t}d\right)}^{2}$ squares. Using the TDMA to schedule the transmission, that is each square takes a turn on the transmission over ${\left(k_{t}d\right)}^{2}$ slots, in each slot, a transmitter can send packets to a receiver located at most *d* squares away. In Figure 4, the larger square around a transmitting square is the secrecy zone, which consists of squares that are at most $k_{e}d$ squares away. We firstly establish an achievable secure rate on a single hop.

**Theorem 6.** *In each square, the secrecy rate that a legitimate source-destination pair can obtain is $R_{s}\left(d\right)=\Omega \left(d^{-\alpha -2}\right)$, if:*

(17) $$\frac{\left(N_{0}+c^{*}\right)d^{-\alpha }}{N_{0}{\left(d+1\right)}^{-\alpha }}<k_{e}^{\alpha }$$

where $c^{*}$ is a constant and d is the transmission range.

**Proof.** Assuming that transmitter *i* in square $s_{i}$ transmits toward destination *j* located in square $s_{j}$ at a distance of *d* squares away, we obtain the SINR of the legitimate receiver as follows:

(18) $$SINR_{ij}=\frac{P_{i}d_{ij}^{-\alpha }}{N_{0}+\Sigma_{\zeta \in T}P_{\zeta }d_{\zeta j}^{-\alpha }}$$

where $d_{ij}$ is the distance between source node *i* and destination node *j*, and $d_{\zeta j}$ is the distance between interferer ζ∈T and the receiver.

For the case of eavesdropper e∈ε, the upper bound SINR at the eavesdropper is:

(19) $$SINR_{e}\leq \frac{P_{i}d_{ie}^{-\alpha }}{N_{0}}$$

where $d_{ie}$ is the distance between the transmitter and eavesdropper *e*, where the upper bound of $SINR_{e}$ is obtained by getting rid of the interference at the eavesdropper. Note that the distance between the *i* and *j* is at most $\frac{2\pi r_{i}}{x}\left(d+1\right)$, *i.e.*,

(20) $$d_{ij}\leq \left(d+1\right)\frac{2\pi r_{i}}{x}$$

and:

(21) $$d_{ie}\leq k_{e}d\frac{2\pi r_{i}}{x}$$

Let I(d) denote the upper bound of the interference caused by simultaneous transmitter nodes. Then,

(22) $$\begin{array}{cc} I(d) & \leq \sum_{\zeta =1}^{\infty }8\zeta P_{i+\zeta k_{t}d}{\left(\frac{1}{\sqrt{2}r_{i+\zeta k_{t}d}-r_{i}}\right)}^{\alpha }\\ & \leq \sum_{\zeta =1}^{\infty }8\zeta P_{i+\zeta k_{t}d}{\left(\frac{1}{\sqrt{2}\zeta k_{t}dr_{i}\frac{2\pi }{x}}\right)}^{\alpha }\\ & =\sum_{\zeta =1}^{\infty }8\zeta P_{0}{\left(\frac{1}{\sqrt{2}\zeta k_{t}d}{\left(1+\frac{2\pi }{x}\right)}^{k_{t}\zeta d}\right)}^{\alpha }\\ & =P_{0}{\left(\sqrt{2}k_{t}d\right)}^{-\alpha }\sum_{\zeta =1}^{\infty }8\zeta^{1-\alpha }{\left(1+\frac{2\pi }{x}\right)}^{k_{t}\zeta d\alpha } \end{array}$$

notice that this sum will converge to a constant $c^{*}$, if α>2, and the proof is shown in Appendix A.

Substitute Equation (20)–Equation (22) in Equation (18) and Equation (19); we obtain that:

(23) $$SINR_{ij}\geq \underline{SINR_{ij}}≜\frac{P_{i}{\left(\left(d+1\right)\frac{2\pi r_{i}}{x}\right)}^{-\alpha }}{N_{0}+c^{*}}$$

and:

(24) $$SINR_{e}\leq \bar{SINR_{e^{*}}}≜\frac{P_{i}{\left(k_{e}d\frac{2\pi r_{i}}{x}\right)}^{-\alpha }}{N_{0}}$$

Hence, $SINR_{ij}>SINR_{e}$ for every eavesdropper *e*, if we choose $k_{e}$ such that:

(25) $$\frac{\left(N_{0}+c^{*}\right)d^{-\alpha }}{N_{0}{\left(d+1\right)}^{-\alpha }}<k_{e}^{\alpha }$$

According to the Gaussian wiretap channel capacity [27], the secrecy rate $R_{s}\left(d\right)$ in each square is:

(26) $$R_{s}\left(d\right)=\frac{1}{{\left(k_{t}d\right)}^{2}}\left[\log\left(1+\underline{SINR_{ij}}\right)-\log\left(1+\bar{SINR_{e^{*}}}\right)\right]=\Omega \left(d^{-\alpha -2}\right)$$

where $\frac{1}{{\left(k_{t}d\right)}^{2}}$ is the time utilization factor. ☐

Now, similar to Lemma 2 in [22], we adopt the multi-hop randomization strategy, which guarantees the security over the entire path, from source to destination, at each eavesdropper listening to all transmissions. In [22], the authors assumed that each legitimate node used identical power for transmission, while we assign different powers for different nodes, as shown in Lemma 5. Despite the difference in the power, the proof goes along the same line as [22]. For conciseness, we omit details.

**Lemma 7.** *(Lemma 2 in [22]) If we can secure each hop from an eavesdropper, then we can ensure secure for all hops from any eavesdropper located on the edge of the secrecy zone.*

In Section 4, we have described the circular percolation model and constructed highways without the constraint of security. If taking the secrecy constraint into consideration, a square is open if the square contains at least one legitimate node and there is no eavesdropper within the secrecy zone of the square. The following result gives the existence of a sufficient number of secure highways in intra-cluster transmission.

**Lemma 8.** *We can construct a number of $\Theta \left(\sqrt{\frac{n^{1-v}}{(1-v)\log n}}\right)$ radial secrecy highways and $\Theta \left(\frac{\log(\log n)}{\delta -2}\sqrt{\frac{n^{1-v}}{\log n}}\right)$ encircling highways within the radius $\rho_{\max}$, if $\lambda_{e}=o\left({\left(\log n\right)}^{-\frac{\delta }{\delta -2}}\right)$.*

**Proof.** For a given square, let $q_{i}$ be the probability of $s_{i}$ contained in a secrecy zone without eavesdroppers. According to a Poisson random distribution, the average number of eavesdroppers located in a secrecy zone is $\lambda_{e}{\left(2k_{e}d+1\right)}^{2}{\left(\frac{2\pi }{x}r_{i}\right)}^{2}$, and $q_{i}$ can be denoted as:

(27) $$q_{i}=e^{-\lambda_{e}{\left(2k_{e}d+1\right)}^{2}\frac{n}{m}{\left(\frac{2\pi }{x}r_{i}\right)}^{2}}$$

Since $r_{i}<\rho_{\max}=\Theta \left({\left((1-v)\log n\right)}^{\frac{1}{\delta -2}}\right)$, n→∞, we have that $q_{i}$ trends to one if $\lambda_{e}=o\left({\left(\log n\right)}^{-\frac{\delta }{\delta -2}}\right)$.

Note that the status of edges in squares is not statistically independent due to the intersection of the associated secrecy zone. If both secrecy zones did not cross, the states of two squares would be independent. This occurs when the squares are at a distance of at least $2k_{e}d$ squares away. Thus, we can conclude that the dependent model is related to a finite dependence model, as $k_{e}$ and *d* are constants. According to Theorem 7.65 in [28], this dependent model stochastically dominates an independent model. Let $p_{i}^{\prime }$ be the probability that squares are independently open. If $p_{i}q_{i}$ can be made arbitrarily high, $p_{i}^{\prime }$ will be close to one. Therefore, under the assumption of the finite dependence model and some desirable properties, we can prove that the network will still percolate with the same properties, since both $p_{i}$ and $q_{i}$ can be set sufficiently large.

Under the independent square model, by Theorem 5 in [6], with a square openness probability of $p_{i}^{\prime }$, we can obtain that there are $\Theta \left(\sqrt{\frac{n^{1-v}}{(1-v)\log n}}\right)$ radial secrecy highways and $\Theta \left(\frac{\log(\log n)}{\delta -2}\sqrt{\frac{n^{1-v}}{\log n}}\right)$ annuli highways. ☐

Till now, we have established the existence of a sufficient number of secure highways using the circular percolation model. Since there are four phases for packet transmission, we will derive the secrecy rate in each phase to find the rate bottleneck.

**Lemma 9.** *If a cluster is divided into w sectors with an arc of 2π/w, then for each sector $SR_{i}$, there are no more than $\frac{2n/m}{w}$ legitimate nodes located.*

**Proof.** Let $|SR_{i}|$ represent the number of legitimate nodes located in $SR_{i}$ and $P_{w}$ be the probability that there exists a sector containing more than $\frac{2n/m}{w}$ nodes. For each sector, the number of nodes follows a Poisson distribution of $\frac{2n/m}{w}$. According to the Chernoff bound, when n→∞, then:

(28) $$\begin{array}{cc} P_{w} & \leq wP\left(|SR_{i}|>\frac{2n/m}{w}\right)\\ & \leq we^{-\frac{n/m}{w}}{\left(\frac{e^{\frac{n/m}{w}}}{\frac{2n/m}{w}}\right)}^{\frac{2n/m}{w}}\\ & =we^{-\frac{n/m}{w}}{\left(\frac{e}{2}\right)}^{\frac{2n/m}{w}}\to 0 \end{array}$$

Therefore, we can get that there is no sector existing with more than $\frac{2n/m}{w}$ nodes. ☐

**Lemma 10.** *In Phase 1, if the density of the eavesdropper is $\lambda_{e}=o\left({\left(\log n\right)}^{-\frac{3\delta -4}{\delta -2}}\right)$, each legitimate node can achieve a secrecy access rate $R_{1}=\Omega \left({\left(\log\left(\sqrt{\left(1-v\right)n^{1-v}\log n}\right)\right)}^{-3-\alpha }\right)$ with a node on the radial highway.*

**Proof.** According to Theorem 5 in [6], if we choose *ϵ* and *κ* appropriately, there exist at least $\Omega (\log\left(\frac{n/m}{x}\right))$ radial highways within a sector of arc $\frac{2\pi }{x}\left[\kappa \log\left(\frac{n/m}{x}\right)-\epsilon \right]$. From percolation theory, we know that each highway may not be fully contained in its corresponding sector, and it may deviate from it. However, it never deviate by an arc of $\frac{2\pi }{x}\left[\kappa \log\left(\frac{n/m}{x}\right)-\epsilon \right]$ from its corresponding sector, *i.e.*, it will not be father than $\kappa \log\left(\frac{n/m}{x}-\epsilon \right)$ squares.

By Theorem 6, let $d=\kappa \log\left(\frac{n/m}{x}-\epsilon \right)$; we can get that the secrecy rate between a legitimate node and an access node is:

(29) $$R\left(\kappa \log\left(\frac{n/m}{x}-\epsilon \right)\right)=\Omega \left({\left(\sqrt{\log(\left(1-v\right)n^{1-v}\log n)}\right)}^{-2-\alpha }\right)$$

Since there is an amount of nodes in a square, they need to share the bandwidth. From Lemma 9, we have that, if the associated secrecy zone contains no eavesdropper, the secrecy rate for Phase 1 is $\Omega \left({\left(\sqrt{\log(\left(1-v\right)n^{1-v}\log n)}\right)}^{-3-\alpha }\right)$. Next, we elaborate that this will happen if $\lambda_{e}=o\left({\left(\log n\right)}^{-\frac{3\delta -4}{\delta -2}}\right)$ as *n* goes to infinity.

For the *i*-th annulus, the area of the guard zone is $A_{i}={\left(2k_{e}d+1\right)}^{2}{\left(\frac{2\pi }{x}r_{i}\right)}^{2}$, which is the area to eliminate the eavesdroppers. Let |ε| be the number of eavesdroppers in a cluster (Poisson with parameter $\frac{\lambda_{e}n}{m}$) and |L| as the total amount of legitimate nodes in a cluster. In addition, we denote the total area that the eavesdroppers make it impossible for a legitimate user to arrive at a highway as $A_{\varepsilon }$. Clearly, $A_{\varepsilon }\leq A_{\max}\left|\varepsilon \right|$, where $A_{\max}={\left(2k_{e}d+1\right)}^{2}{\left(\frac{2\pi }{x}\rho_{\max}\right)}^{2}$. For each $A_{i}$, let the amount of legitimate nodes in this region be $L_{i}$. According to the heterogeneity of node distribution, we have $L_{i}\leq \frac{n}{m}r_{i}^{-\delta }A_{i}$. Thus, for each cluster, by the Chebyshev inequality, we have:

$$\left|\varepsilon \right|\leq \left(1+\epsilon \right)\lambda_{e}\frac{n}{m}$$

$$\left|L\right|\geq \left(1-\epsilon \right)\frac{n}{m}$$

(30) $$L_{A_{\max}\left|\varepsilon \right|}\leq \left(1+\epsilon \right)\frac{n}{m}A_{\max}\left|\varepsilon \right|$$

for any ϵ∈(0,1) with high probability as n→∞. Let *ϝ* be the fraction of legitimate nodes that cannot transmit to highways due to the eavesdropper, and we can obtain the upper bound of *ϝ* as:

(31) $$ϝ\leq \frac{L_{A_{\max}\left|\varepsilon \right|}}{L}\leq \frac{{\left(1+\epsilon \right)}^{2}{\left(2k_{e}d+1\right)}^{2}\frac{n}{m}{\left(\frac{2\pi }{x}\rho_{\max}\right)}^{2}\lambda_{e}\frac{n}{m}}{\left(1-\epsilon \right)\frac{n}{m}}\to 0$$

with the probability going to one as n→∞. The first inequality is deduced from the intersecting secrecy zones caused by eavesdroppers, and the second inequality derives from Equation (30), while the limit holds as $d=\kappa \log\left(\frac{n/m}{x}-\epsilon \right)$ and $\lambda_{e}=o\left({\left(\log n\right)}^{-\frac{3\delta -4}{\delta -2}}\right)$. Under this condition, we can conclude that almost all of the legitimate nodes are securely connected to the highways as n→∞. ☐

Phase 4 is the opposite process of Phase 1. Therefore, a similar conclusion can be made for this phase.

**Lemma 11.** *In Phase 4, if the density of eavesdropper $\lambda_{e}=o\left({\left(\log n\right)}^{-\frac{3\delta -4}{\delta -2}}\right)$, then a legitimate node can receive information securely from the highway at a rate of $R_{4}=\Omega \left({\left(\log\left(\sqrt{\left(1-v\right)n^{1-v}\log n}\right)\right)}^{-3-\alpha }\right)$.*

In the highway phase, the information is transmitted hop by hop. Let d=1 in Theorem 6; we obtain the secrecy rate in Phase 2.

**Lemma 12.** *In Phase 2, if the density of eavesdropper $\lambda_{e}=o\left({\left(\log n\right)}^{-\frac{\delta }{\delta -2}}\right)$, then a legitimate node on the radial highway can achieve a secrecy rate $R_{2}=\Omega \left(\frac{1}{\sqrt{n^{1-v}\left(1-v\right)\log n}}\right)$.*

**Proof.** According to the highway system, the transmission is occurring from one square to a neighboring square, where within the secrecy zone, there are no eavesdroppers. Thus, using Theorem 6 and letting d=1, the secrecy rate in Phase 2 is Ω(1). Since $x>>\log\frac{n}{mx}$, there are Ω(x) radial paths extended from internal to external. By Lemma 9, we have that there are at most $\frac{2n}{mx}$ users w.h.p. in the sector of $\frac{2\pi }{x}$. That is, each node can enjoy a rate of order $\Omega \left(\frac{1}{\sqrt{n^{1-v}\left(1-v\right)\log n}}\right)$ in the radial highway. ☐

In this way, the following lemma gives the secrecy rate of Phase 3.

**Lemma 13.** *The legitimate nodes on the annulus highway can enjoy a per-node secrecy rate $R_{3}=\Omega \left(\sqrt{\frac{(1-v)\log n}{n^{1-v}}}\cdot f\left(\delta \right)\right)$, where f(δ) is a “heterogeneous factor”, which is only decided by δ.*

**Proof.** Compared to the radial highways, it is more complicated for data delivered around the cluster, since the number of nodes served by the annulus paths is not identical. Assume each annulus highway is identical, similar to Lemma 12; the secrecy rate of order $\Omega \left(\sqrt{\frac{(1-v)\log n}{n^{1-v}}}\right)$. Nevertheless, due to the impact of heterogeneity, we denote the achievable secrecy rate as $\Omega \left(\sqrt{\frac{(1-v)\log n}{n^{1-v}}}\cdot f\left(\delta \right)\right)$, where f(δ) is a function of heterogeneous factor *δ*, which will be discussed in detail in Appendix B. ☐

Comparing the secrecy rate and the tolerable density of eavesdroppers in each phase, the secrecy rate of intra-cluster transmission can be concluded as follows.

**Theorem 14.** *For the intra-cluster traffic, if the density of eavesdropper $\lambda_{e}=o\left({\left(\log n\right)}^{-\frac{3\delta -4}{\delta -2}}\right)$, then each legitimate node located within $\rho_{\max}$ can achieve a secrecy rate of $R_{s}^{intra}=\Omega \left(\frac{1}{\sqrt{n^{1-v}\left(1-v\right)\log n}}\right)$,*

**Proof.** Comparing the achievable secrecy rate in each phase, the rate bottleneck occurs in Phase 2. Since the information is transmitted hop by hop, we need to guarantee security in each phase. Thus, comparing the density of eavesdroppers in each phase, we can get that, if the density of eavesdroppers $\lambda_{e}=o\left({\left(\log n\right)}^{-\frac{3\delta -4}{\delta -2}}\right)$, the secrecy rate of intra-cluster transmission is $R_{s}^{intra}=\Omega \left(\frac{1}{\sqrt{n^{1-v}\left(1-v\right)\log n}}\right)$ (the proof is in Appendix B). ☐

## 6. The Secrecy Transmission of Inter-Cluster Traffic

**Figure 5:**
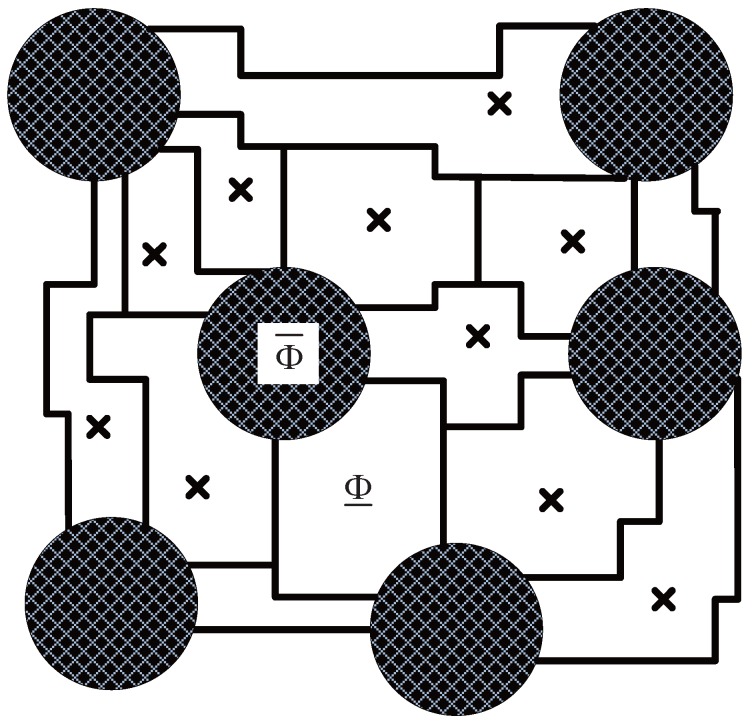
An illustration of information pipelines among clusters. Since the minimum node density is $\underline{\Phi }$, we can construct $\Omega (\sqrt{A\underline{\Phi }})$ pipelines among clusters. The crosses denote the eavesdroppers.

Since we focus on the cluster-sparse network, the node density outside the circular square is much lower. Thus, we cannot use the highway system constructed in Section 5. However, according to the distribution of PPP, we can extract part of nodes with a density of *ϕ* to build “information pipelines”, where *ϕ* is smaller than $\underline{\Phi }$ and is selected randomly and uniformly. As shown in Figure 5, we use these “information pipelines” to connect the clusters.

Similarly, by virtue of percolation theory, we can divide the area into regular squares with a side length of $\sqrt{c_{0}/\underline{\Phi }}$, where $c_{0}$ is a constant. For some special large value $c_{0}$, we can extract part of nodes to form “information pipelines”, which is similar to the highways constructed in the homogeneous network. However, due to the heterogeneity, each square contains difference nodes.

In the previous section, we have already achieved the secrecy rate in the cluster area through a highway system. Within the dense areas, information is transmitted by the routes formed by the highway system. Only if the destination is located in different clusters, the information will be delivered through the “information pipelines”. Similar to the derivation of intra-cluster transmission, the achievable secrecy rate of inter-cluster traffic can be obtained easily.

Borrowing the tools from percolation theory in [6], we can construct $\Omega (\sqrt{A\underline{\Phi }})$ pipelines among clusters. All of these pipelines need to serve Θ(m) clusters. Similar to Lemma 1 in [22], a secrecy zone is employed to protect the secrecy transmission over a single hop, where the edge of the secrecy zone is not *c*, but $\sqrt{\frac{c_{0}}{\underline{\Phi }}}$. Correspondingly, we can build $\Omega \left(\frac{\sqrt{A\underline{\Phi }}}{m}\right)$ pipelines between two neighboring clusters, *i.e*., each cluster can enjoy $\Omega \left(\frac{\sqrt{A\underline{\Phi }}}{m}\right)$ pipelines. By Theorem 6 in [22], we can conclude that, if $\lambda_{e}=o\left({\left(\log\left(n\underline{\Phi }\right)\right)}^{-2}\right)$, the secrecy rate of inter-cluster transmission is $\Omega \left(\frac{\sqrt{A\underline{\Phi }}}{n}\right)$.

**Theorem 15.** *For the inter-cluster transmission, the achievable secrecy rate is $R_{s}^{inter}=\Omega \left(\frac{\sqrt{A\underline{\Phi }}}{n}\right)=\Omega \left(\sqrt{\frac{\underline{\Phi }}{n}}\right)$, if the density of the eavesdropper $\lambda_{e}=o\left({\left(\log\left(n\underline{\Phi }\right)\right)}^{-2}\right)$.*

**Figure 6:**
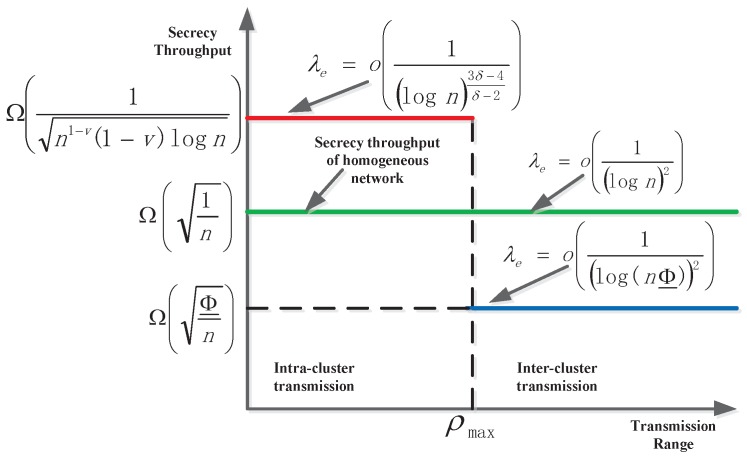
An illustration of secrecy throughput. We also give a comparison with that in a homogeneous networks [22].

Figure 6 compares the throughput under homogeneous networks and heterogeneous networks. By observing the secrecy rate of intra-cluster and inter-cluster transmission, we find that the secrecy rate of intra-cluster transmission is higher than that of homogeneous networks; therefore, the bottleneck occurs in the inter-cluster transmission. This is due to the reduction of the number of highways caused by the lower density and, thereby, the amount of relaying traffic increasing. Particularly, when the network is transferred to a homogeneous network, *i.e.*, $\underline{\Phi }=\Theta \left(\bar{\Phi }\right)=\Theta \left(1\right)$, the secrecy rate is $R_{s}^{inter}=\Omega \left(\frac{1}{\sqrt{n}}\right)$, and the tolerable density of the eavesdropper is $\lambda_{e}=o\left({\left(\log n\right)}^{-2}\right)$, which is the same result as that in homogeneous wireless networks [22].

## 7. Conclusions

In this work, we study the impact of heterogeneity and the secrecy constraint on the capacity of wireless networks. Borrowing the tools from percolation theory, we first constructed a secrecy highway system for intra-cluster and inter-cluster transmission, respectively. With the protection of the secrecy zone, the relationship between secrecy capacity and the tolerable density of the eavesdropper is studied. It is shown that the intra-cluster transmission not only can achieve a higher secrecy capacity, but also can tolerate more eavesdroppers. Moreover, the highway system we constructed is suitable for non-uniform traffic networks, typically, such as social networks. Thus, this work provides an insight model to analyze social networks. Finally, we do not consider the case of eavesdroppers collaborating with each other. Thus, it is a valuable future work to study the scenario of colluding eavesdroppers, where the distribution of legitimate nodes and eavesdroppers will influence the secrecy throughput greatly.

## Appendix

### A. The Proof of Theorem 1

Proof: We prove the summation of Equation (22) to converge to some constant. Since we consider the scenario of n→∞, Equation (22) can be simplified as $\sum_{\zeta =1}^{\infty }\zeta^{1-\alpha }{\left(1+2\pi /x\right)}^{k_{t}\zeta d\alpha }$. Firstly, we solve ${\left(1+2\pi /x\right)}^{k_{t}\zeta d\alpha }$, where *x* is given in Lemma 2. Thus, we obtain:

(A1) $$\begin{array}{cc} {\left(1+2\pi /x\right)}^{k_{t}\zeta d\alpha } & ={\left(1+\frac{2\pi }{\sqrt{\frac{n^{1-v}}{(1-v)\log n}}}\right)}^{\frac{\sqrt{\frac{n^{1-v}}{(1-v)\log n}}}{2\pi }\cdot \frac{2\pi }{\sqrt{\frac{n^{1-v}}{(1-v)\log n}}}\cdot k_{t}\zeta d\alpha }\\ & =e^{\frac{2\pi }{\sqrt{\frac{n^{1-v}}{(1-v)\log n}}}\cdot k_{t}\zeta d\alpha } \end{array}$$

when $d=o(\sqrt{\frac{n^{1-v}}{(1-v)\log n}})$ and n→∞. Then, ${\left(1+2\pi /x\right)}^{k_{t}\zeta d\alpha }=e^{\frac{2\pi }{\sqrt{\frac{n^{1-v}}{(1-v)\log n}}}\cdot k_{t}\zeta d\alpha }\to \Theta \left(1\right)$. Since α>2, the summation Equation (22) converges to a constant.

### B. The Proof of Theorem 2

Proof: The achievable rate of the annulus highway in the intra-cluster phase is derived as follows: According to four phases of the routing scheme, we give a comparison of the secrecy rate on the radial highway and the annulus highway. The secrecy rate on the radial highway can be obtained easily (Lemma 12). Hence, We only need to derive the secrecy rate of the annulus highway. Due to the number of nodes on different annuli not being identical, the derivation of the annulus highway is more complicated than the case of the radial highway. According to the distribution of legitimate nodes, let $E(N_{i})$ be the average number of nodes in annulus *i*. Then, the expectation of $E(N_{i})$ is:

(B1) $$E\left(N_{i}\right)=\frac{n}{m}{\left(r_{i}\frac{2\pi }{x}\right)}^{2}\frac{1}{r_{i}^{\delta }}x$$

Using Equation (7) to substitute $r_{i}$, we can get:

(B2) $$\begin{array}{cc} E(N_{i}) & =\frac{n}{m}{\left(r_{i}\frac{2\pi }{x}\right)}^{2}\frac{1}{r_{i}^{\delta }}x\\ & =4\pi^{2}\frac{n}{m}x^{-1}r_{i}^{2-\delta }\\ & =4\pi^{2}\frac{n}{m}x^{-1}{\left(1+\frac{2\pi }{x}\right)}^{i(2-\delta )}\\ & =4\pi^{2}\frac{n}{m}\sqrt{\frac{(1-v)\log n}{n^{1-v}}}{\left(1+\frac{2\pi }{\sqrt{\frac{n^{1-v}}{(1-v)\log n}}}\right)}^{i(2-\delta )} \end{array}$$

Since δ>2, $E(N_{i})$ is decreased with the increasing of *i*. Therefore, the annulus highway near the center will service the most nodes. As a consequence, the achievable secrecy rate on the annulus highway is:

(B3) $$R_{3}>R_{r}\left(i=1\right)=\Theta \left(\sqrt{\frac{1}{\left(1-v\right)n^{1-v}\log n}}{\left(1+\frac{2\pi }{\sqrt{\frac{n^{1-v}}{(1-v)\log n}}}\right)}^{\left(\delta -2\right)}\right)$$

By comparing the rate $R_{2}$ and $R_{3}$, we can find that $R_{2}<R_{3}$, for δ>2, *i.e.*, the secrecy rate bottleneck is in the phase of the radial highway.
